# Chronic Glomerulonephritis and Malignant Hypertension With PRES (Posterior Reversible Encephalopathy Syndrome) Presenting As Status Epilepticus: A Case Report

**DOI:** 10.7759/cureus.43902

**Published:** 2023-08-22

**Authors:** Kashish Khurana, Sourya Acharya, Samarth Shukla, Sunil Kumar, Preeti Mishra

**Affiliations:** 1 Department of Medicine, Jawaharlal Nehru Medical College, Datta Meghe Institute of Medical Science, Wardha, IND; 2 Department of Pathology, Jawaharlal Nehru Medical College, Datta Meghe Institute of Medical Science, Wardha, IND

**Keywords:** nephrotic syndrome, status epilepticus, posterior reversible encephalopathy syndrome (pres), chronic glomerulonephritis, hypertension

## Abstract

Hypertension risk is a common complication of chronic glomerulonephritis (GN), which includes focal segmental glomerulosclerosis and proliferative forms of GN such as IgA nephropathy. The clinical-radiological phenomenon known as posterior reversible encephalopathy syndrome (PRES) is frequently linked to renal disorders, particularly chronic kidney disease and hypertension. PRES is an acute clinical condition characterized by multiple neurological symptoms such as seizures, impaired consciousness, headaches, visual abnormalities, nausea, and vomiting. In this case report, we discuss status epilepticus due to PRES in a 20-year-old girl who presented with nephrotic syndrome after renal biopsy chronic GN was confirmed. Repeated neuroimaging performed following proper blood pressure management revealed that the lesions had vanished, supporting the diagnosis of PRES. Presumably, PRES remained for 5-7 days in our case. Nephrologists must be familiar with the atypical characteristics of PRES as it is frequently associated with kidney disease. Prompt identification and care prevent irreparable consequences and pointless investigations.

## Introduction

Hypertension is a component linked invariably with chronic glomerulonephritis (GN). Chronic GN patients' hypertension is predominantly volume-dependent, and this rise in blood volume is unrelated to the decline in renal function. As renal damage, including arteriolosclerosis, worsens in patients with chronic GN, salt intolerance develops, and the resulting renal ischemia stimulates the intrarenal renin-angiotensin-aldosterone system (RAAS). In chronic GN, excessive sympathetic nervous system activity also contributes to hypertension. The Kidney Disease Improving Global Outcomes (KDIGO) recommendation states that in patients with chronic renal disease who do not have albuminuria, the target blood pressure should be 140 mmHg systolic and 90 mmHg diastolic. A lower aim of 130 mmHg systolic and 80 mmHg diastolic is advised for the majority of individuals with an albumin excretion rate of 30 mg/24 hours (i.e., those with both micro- and macroalbuminuria). In all individuals with an albumin excretion rate of less than 30 mg per 24 hours, the use of medications that block the RAAS system is advised or encouraged [1].

In individuals with chronic GN, the combination of a RAAS blockade, a calcium channel blocker, and a diuretic may be beneficial in lowering urine protein excretion and achieving the target blood pressure [1].

Posterior reversible encephalopathy syndrome (PRES) is frequently found in patients with kidney disorders, particularly chronic kidney disease. Characteristic imaging changes due to vasogenic oedema are predominantly seen in the parieto-occipital white matter of the brain. Although the pathophysiology of PRES is not fully understood, certain theories are suggested for understanding it. The most popular theory is that acute hypertension may be associated with dysfunction of cerebral autoregulation, causing the breakdown of the blood-brain barrier and leading to extravasation from vessels into the brain parenchyma. Poor sympathetic innervation in the posterior circulation predisposes predilection for brain oedema in this region. These unusual characteristics could lead to a delay in diagnosis and treatment if they are mistaken for other etiologies [2].

Visual disturbance, headache, seizures, and diminished consciousness are just a few of the many clinical signs that can accompany PRES. Oedema is visible on magnetic resonance imaging (MRI) and typically affects the posterior subcortical areas. Hypertension, pre-eclampsia/eclampsia, renal failure, cytotoxic chemicals, and autoimmune diseases are some of the triggers associated with it. Although the exact cause of PRES is unknown, endothelial dysfunction is thought to be a contributing factor. PRES is not always reversible and may be linked to significant morbidity and even mortality, even though the majority of patients recover [1].

The clinical-radiological diagnosis of PRES is made on the basis of a combination of clinical features, risk factors, and results from MR brain scans. Multiple neurological symptoms may appear or they may manifest alone, and they may change over the course of the acute stage of the illness. It typically manifests as a combination of nausea, one or multiple episodes of seizures, decreased mental function, headaches, and vision loss, but it can also include additional localised abnormalities like weakness, sensory disturbances, or speech disturbances [2].

The PRES has a variety of underlying causes and may be brought on by medical interventions (such as antineoplastic therapy) or by a PRES-related medical condition (such as autoimmune disorders or eclampsia).

PRES can also present as status epilepticus. Current definitions of status epilepticus, a neurological emergency, include recurrent seizures without recovery in between them or clinical or electrographic seizure activity lasting longer than 5 minutes [1]. Convulsive, non-convulsive, focal, or myoclonic seizure activity are all possible. Early access to the right care can stop morbidity and mobility, including permanent brain injury. Refractory status epilepticus is a form of condition that has a poor prognosis and does not react to the recommended antiepileptic medications. Status epilepticus can have a variety of aetiologies, such as a recognised epilepsy problem or other seizure disorders that are brought on by a systemic illness. Common causes include infections of the central nervous system, metabolic abnormalities, head injuries, toxic substances, hypoxia, and hypertensive emergencies [3].

Hypertension is the most important risk factor of PRES, a rare clinic-radiological condition that is thought to be a close differential diagnosis of hypertensive encephalopathy. Patients who are treated for nephrotic syndrome with extended steroid therapy or calcineurin inhibitor medication are at risk of developing PRES [1]. As a result of the low index of suspicion, early diagnosis and treatment of the syndrome are essential for the resolution of the symptoms and radiological features as well as to prevent complications like long-term neurological deficit [2].

## Case presentation

A 20-year-old female with no previous significant medical or family history presented to the emergency department with complaints of 5-6 episodes of generalised tonic-clonic seizures for 2-3 hours with each episode lasting for around 5-6 minutes. The patient did not regain consciousness during the interictal period. It was associated with frothing from the mouth. The patient was unconscious and responsive to deep pain stimuli, she was intubated in the emergency department to protect her airway and was given a loading dose of anti-epileptic injection of lorazepam 4 mg stat and injection of levetiracetam 1 gram stat.

On general examination, her blood pressure was found to be raised (210/120 mm Hg) for which she was given an injection of furosemide 40mg and an injection of labetalol 10 mg intravenous stat. After the stabilisation of the patient, an MRI brain was done and she was shifted to the medicine intensive care unit where she was evaluated further.

On further examination, her blood pressure was still found to be raised (190/110 mm Hg) and her heart rate was 120 beats per minute, pallor was present, and icterus, pedal oedema, cyanosis, and clubbing were absent. On systemic examination, the patient had a Glasgow coma score of 8, was drowsy, responding to deep pain stimuli, bilateral plantar was extensor, and bilateral pupils were reactive to light. Respiratory examination revealed bilateral equal air entry with no crepitations, heart sounds heard with no murmurs and the abdomen was soft and non-tender. In view of uncontrolled blood pressure, she was started on intravenous nitroglycerine infusion. Which was slowly tapered off within 24 hours and she was switched to oral nicardipine 10 mg thrice daily, oral metoprolol 25 mg twice daily and oral furosemide 40 mg twice daily.

**Figure 1:**
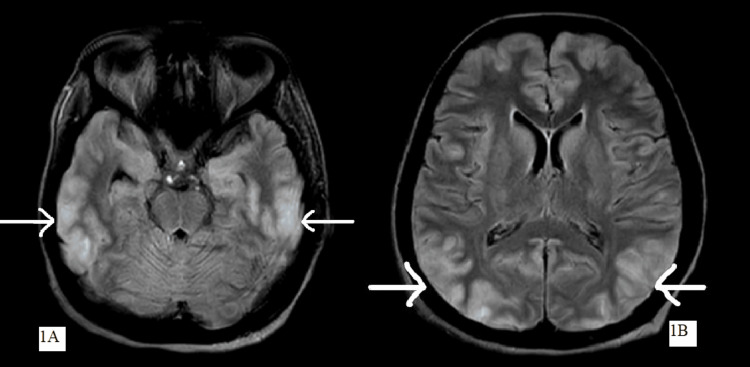
MRI brain showing altered signal intensity with vasogenic oedema in bilateral frontal, temporal and parietal lobes (as shown in Figure 1A) and occipital lobes (as shown in Figure 1B) suggestive of posterior reversible encephalopathy syndrome. MRI: magnetic resonance imaging

MRI brain was suggestive of altered signal intensity with vasogenic oedema in bilateral frontal, temporal, parietal and occipital lobes appearing hyperintense on T2-weighted fluid-attenuated inversion recovery (T2/FLAIR) showing high signal intensity on diffusion-weighted imaging (DWI) suggestive of PRES with hypoplastic left transverse sinus as shown in Figure 1.

All routine investigations were done and were suggestive of severe anaemia (haemoglobin: 6.5 g%), deranged kidney profile (serum urea: 148 mg/dL, serum creatinine: 2.7 mg/dL) with raised inflammatory markers (erythrocyte sedimentation rate (ESR): 42 mm/hour and C-reactive protein (CRP): 51 mg/dL). Table 1 shows all blood investigations. Ultrasound abdomen and pelvis were suggestive of left-sided pleural effusion with minimal ascites with grade III renal parenchymal disease. CT kidney ureter bladder was suggestive of mild free fluid in the abdomen and pelvic cavity. Fundoscopy was done which did not show any signs of hypertensive retinopathy. 2D echocardiography was suggestive of trivial mitral regurgitation, no evidence of mitral stenosis, no regional wall motion abnormality, a thin rim of pericardial effusion, and a left ventricular ejection fraction of 60%.

**Table 1:** Laboratory investigations of the patient

Parameters	Value	Normal Value
Haemoglobin	6.5 g%	12-15 gm %
Total leukocyte count	11600	4000-11000 cu. mm
Platelet count	2.15	1.5-4.1 lakhs/cu. mm
Mean corpuscular volume	67.6	83-101 fl
Alkaline phosphatase	133	38-126 U/L
Alanine aminotransferase	37	<35 U/L
Aspartate aminotransferase	28	14-36 U/L
Serum albumin	2.4	3.5-5 g/dL
Urea	148	9-20 mg/dL
Creatinine	2.7	0.6-1.25 mg/dL
Potassium	5.5	3.5-5.1 mmol/l
Sodium	149	135-150 mmol/l
Calcium	8.9	8.4-10.2 mg/dL
Magnesium	2.0	1.6-2.3 mg/dL
C3 levels	101	80-165 mg/dL
C4 levels	4.72	14-44 mg/dL
Erythrocyte sedimentation rate	42	0-16 mm/hour
C-reactive protein	51	<1 mg/dL
Serum procalcitonin	89.1	< 10 ng/mL
Urine protein/creatinine ratio	180	20-200 mg/gm of creatinine

Further detailed laboratory investigations were sent like anti-nuclear antibody, anti-streptolysin o antibody titre, c-anti neutrophilic cytoplasmic antibody (c-ANCA), p-ANCA, anti-phospholipid antibodies (APLA) and complement levels (C3 and C4 levels) which were found to be negative.

**Figure 2:**
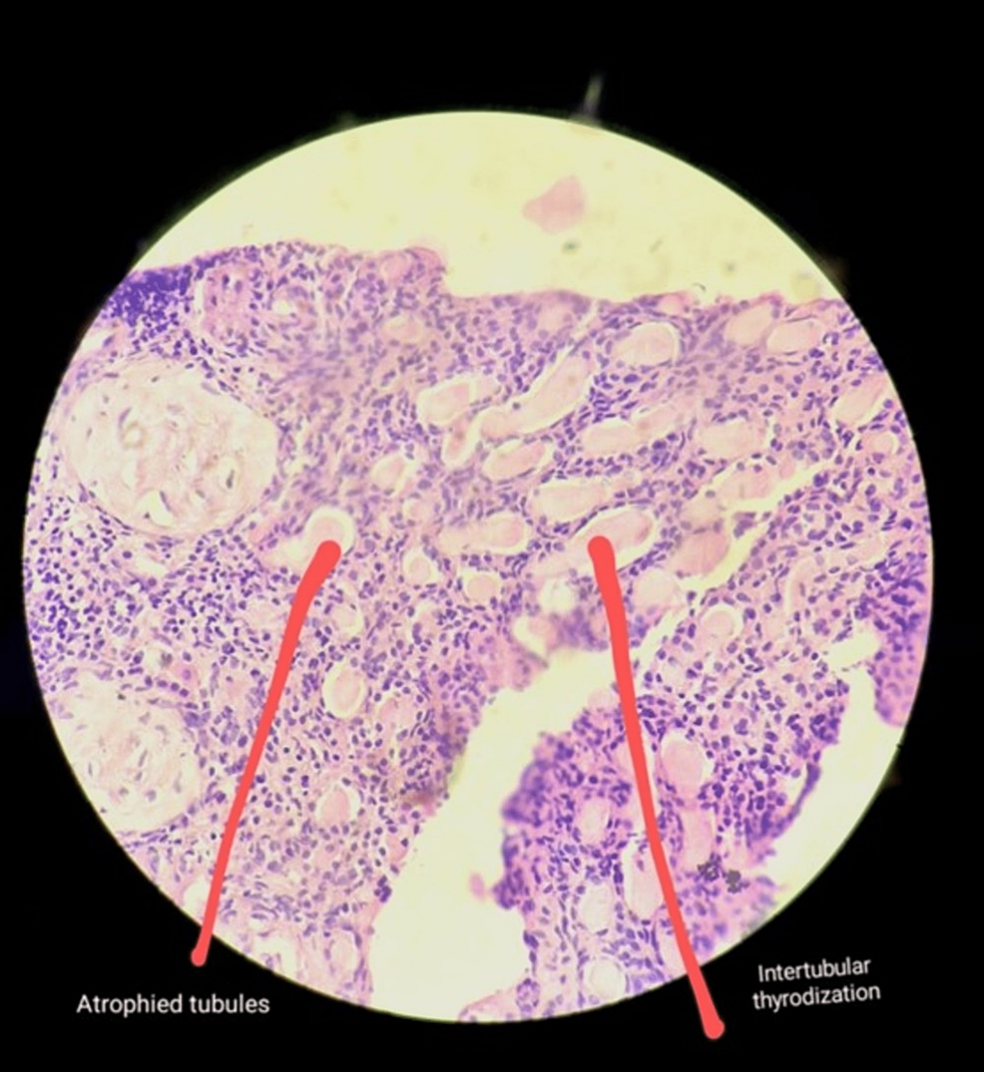
Kidney biopsy showing unremarkable tubules with lumen showing proteinaceous deposits and interstitium showing chronic non-specific lymphoplasmacytic inflammatory infiltrate suggesting chronic glomerulonephritis.

The 24-hour urinary protein was in the nephritic range and significant proteinuria - 1507 mg/24 hours. Urine routine microscopy revealed the presence of 1-2 RBCs per high power field and albuminuria was present. She was then scheduled for a renal biopsy. A kidney biopsy was done which was suggestive of 2-3 glomeruli showing global glomerulosclerosis, unremarkable tubules with lumen showing proteinaceous deposits and interstitium showing chronic non-specific lymphoplasmacytic inflammatory infiltrate suggesting chronic GN as shown in Figure 2. A nephrology opinion was taken and was advised to treat the patient with chronic kidney disease. As the patient’s serum creatinine levels were persistently around 2.5-3 mg/dL, and urea was also not on a rising trend; haemodialysis was not done.

Eventually, as the patient improved clinically and became conscious with no further episodes of seizures, she was extubated and was monitored closely. She was then shifted to the ward when she became stable. She was also weaned off from the intravenous furosemide and labetalol slowly as her blood pressure became stabilized and shifted to oral metoprolol 25 mg extended release with cilnidipine 10 mg once a day. Eventually, she was discharged from the hospital and called for follow-up every two weeks.

## Discussion

Although numerous independent risk factors, including hypertension, autoimmune illness, and immunosuppression are present (sometimes concurrently) in renal disease, little is known about PRES in this condition. In patients with end-stage renal disease (ESRD), PRES could go undiagnosed. In this population, marked hypertension is a major risk factor that is connected to extracellular fluid volume growth. Findings from neuroimaging studies that involve both anterior and posterior circulation areas can be varied. There have been sporadic reports of PRES with kidney disease, but there is a dearth of thorough investigation. In Hinchey's original paper, 80% of the patients had hypertension, and more than 50% of them had renal failure of varied severity [3].

The diagnosis of PRES requires the use of neuroimaging. The posterior cerebral hemispheres often show symmetrical white matter oedema, however, there can be exceptions. There have been reports of anterior cortical lesions, however, they often occur in more serious instances and are frequently accompanied by oedema in the posterior circulation areas [3].

Vasogenic oedema is the PRES's defining symptom. There is an ongoing debate on the role of hypertension, which is frequently cited as the primary cause of vasogenic oedema. There have been put forth two theories. According to the most widely accepted explanation, severe hypertension prevents the cerebral vasculature from regulating itself, causing hyperperfusion, arteriolar dilatation, and vasogenic oedema. A relative absence of sympathetic innervation (because the sympathetic system raises the threshold of autoregulation) has been proposed as an explanation for PRES's preference for the posterior regions of the brain. However, blood pressure is not always increased in PRES, particularly in situations involving organ donation and immunosuppression. Additionally, the severity of hypertension does not always correlate with the level of vasogenic oedema [4].

According to the second idea, the main issue is cerebral vasoconstriction, which causes a capillary leak and downstream hypoperfusion, ischemia, and vasogenic oedema. Consequently, hypertension may not be the only factor causing harm in all cases. Vasospasm and hypoperfusion can be brought on by endothelial cell failure, which is present in autoimmune illnesses and in patients who have had organ transplantation. Vascular endothelial growth factor (VEGF) is secreted by cerebral arteries in response to the resultant hypoxia, which causes them to become more permeable and cause oedema [4].

The use of calcineurin inhibitors and accelerated hypertension are two prevalent instances where PRES is encountered in conjunction with kidney failure. Patients on immunosuppressive regimens do not necessarily need to have hazardous medication levels or hypertension when developing PRES. In light of the typically favourable prognosis, it is a disorder that necessitates early discovery and immediate treatment [3].

There has been much evidence of PRES association with various conditions like postpartum hemolytic uremic syndrome, cortical blindness, hemolytic anaemia, pre-eclampsia, and eclampsia [4-7].

Although it has primarily been discussed in relation to the adult population, paediatrics experience is growing. Children with PRES have been described in several publications; they also had nephrotic syndrome, chronic nephritis, vasculitis, malignancy, acute renal failure, and a history of medication usage. The prevalence of PRES has been noted in all life stages, with a surge in adolescence to middle age, although it can even affect young children and the elderly. The most frequent cause is systemic hypertension, however, there are other aetiologies as well, such as the use of immunosuppressive medications, organ transplants, nephrotic conditions, and sepsis [4].

## Conclusions

Renal disorders especially GN is associated with uncontrolled hypertension. Both renal disorders and hypertension can predispose to PRES which is a medical emergency. Status epilepticus can be the first presentation of PRES as in this case. Any patient presenting with seizures on a background of renal disease should be astutely investigated by neuroimaging to rule out PRES. Aggressive blood pressure management along with targeted therapy for the primary disease should normalise the insult to the brain, as, usually PRES is a reversible condition.
